# Hybrid Mucointeractive Delivery Systems—Alternative Approaches to Mucosal Delivery

**DOI:** 10.3390/molecules31142502

**Published:** 2026-07-17

**Authors:** Konrad Zabielski, Paweł Sajkiewicz, Angelika Zaszczyńska, Tomasz Kowalczyk

**Affiliations:** Laboratory of Polymers & Biomaterials, Institute of Fundamental Technological Research, Polish Academy of Sciences, Pawinskiego 5b St., 02-106 Warsaw, Poland; kzab@ippt.pan.pl (K.Z.); azasz@ippt.pan.pl (A.Z.)

**Keywords:** mucoadhesion, mucopenetration, mucolysis, drug delivery, mucosa, transmucosal, polymers, nanoparticles, nanotechnology

## Abstract

Mucosa can be found in the eyes, oral cavity, bladder, vagina, airways, and gastrointestinal tract. It is an attractive route of administration for systemic or topical delivery of therapeutics. However, the mucus layer acts as a protective barrier, limiting the amount of biomolecules that reach the underlying epithelium. Mucoadhesion, mucodiffusion, and mucolysis are well-established mucointeractive strategies that can improve therapeutic outcomes, but due to their individual limitations, the resulting delivery is often still unsatisfactory. In recent years, drug delivery systems have emerged that combine multiple mucointeractive strategies, which we define here as hybrid mucointeractive delivery systems. This work aims to provide a general overview of such drug delivery systems, which include particle-releasing macrostructures such as gels, foams, films, and fibers, as well as systems such as zeta potential-changing particles and self-emulsifying drug delivery systems. Their potential, possible future, and limitations are discussed as well.

## 1. Introduction

Many different drug administration routes have been developed to date. These routes include enteral (oral, sublingual, buccal), parenteral (intravenous, intramuscular, subcutaneous), intranasal, inhalational, vaginal, transdermal, and others [1]. Each of these strategies has its advantages and limitations that must be considered in clinical practice to provide the best treatment for patients. However, very often, they are still not satisfactory. Developing novel approaches or improving existing ones is thus of utmost importance.

Mucosal delivery has several advantages over other administration routes. First of all, it is a non-invasive approach, and greater patient compliance can be achieved. Secondly, therapeutics can be delivered in a localized manner, decreasing the likelihood of undesired systemic effects. However, systemic delivery is also possible due to the high vascularization of the lamina propria and submucosal layer, but only if a delivery system possesses sufficient permeating properties. Furthermore, mucosal delivery systems can be used to avoid first-pass metabolism and protect therapeutics from enzymatic degradation before reaching the designated site [2]. Additionally, the mucosa can be considered as a viable site for vaccine delivery [3,4]. Moreover, challenges with delivery to the central nervous system due to the blood–brain barrier can be overcome by choosing the nasal mucosa as an administration site, which provides direct delivery to the brain through the olfactory region [5,6].

Out of all delivery routes, the mucosal route is one of the oldest ways for the administration of therapeutics. Medicine taken orally has to pass through the mucosa in the gastrointestinal tract to enter systemic circulation and provide a therapeutic effect. However, the mucosa possesses several protective mechanisms, which hinder the delivery of therapeutics. Only in the early 1980s were the first mucointeractive drug delivery systems presented to improve the efficacy of this approach [7]. A decade later, such formulations were introduced for the first time to the pharmaceutical market. Since then, many different delivery systems have been developed that aimed at improving mucosal drug delivery. Three main approaches can be found in the literature: mucoadhesion, mucodiffusion, and mucolysis. Each of them interacts differently with the mucosa to deliver therapeutics. Mucoadhesion increases residence time through the binding of the polymer with the mucosa. In contrast, mucodiffusion and mucolysis allow for unobstructed passage through mucus by minimizing polymer–mucosa interactions or changing the mucus structure to increase its permeability, respectively. Despite improvements in mucosal delivery, these mucointeractive strategies are limited in overcoming the mucosal barrier. In recent years, several alternative approaches have been presented to address their limitations by combining them.

Many review articles have covered the subject of mucoadhesion, mucodiffusion, and mucolysis. Only a few of them discussed research articles that focused on a combination of these strategies, but even then, they are very often limited only to nanoparticulate systems [8,9,10]. In their review article, Taipaleenmäki et al. discussed cases of small particles incorporated into larger carriers [11]. Similarly, Tran et al. covered a few examples of nanoparticulate systems in mucoadhesive macrostructures for buccal delivery [12]. In 2023, Zhang et al. included a short section that presented eight mucoadhesion-to-mucopenetration delivery systems [13]. One mini-review was also published on this topic [14]. In this review article, we aim to provide an extensive overview of hybrid delivery systems that try to combine multiple mucointeractive strategies in different shapes and forms. The efficacy of such systems and their potential future are discussed here as well. The goal of this review article is to also show that mucosal delivery is not restricted to the three basic mucointeractive strategies, but that more advanced and potentially more promising approaches are available.

## 2. Mucus as a Barrier to the Delivery of Therapeutics

**Figure 1:**
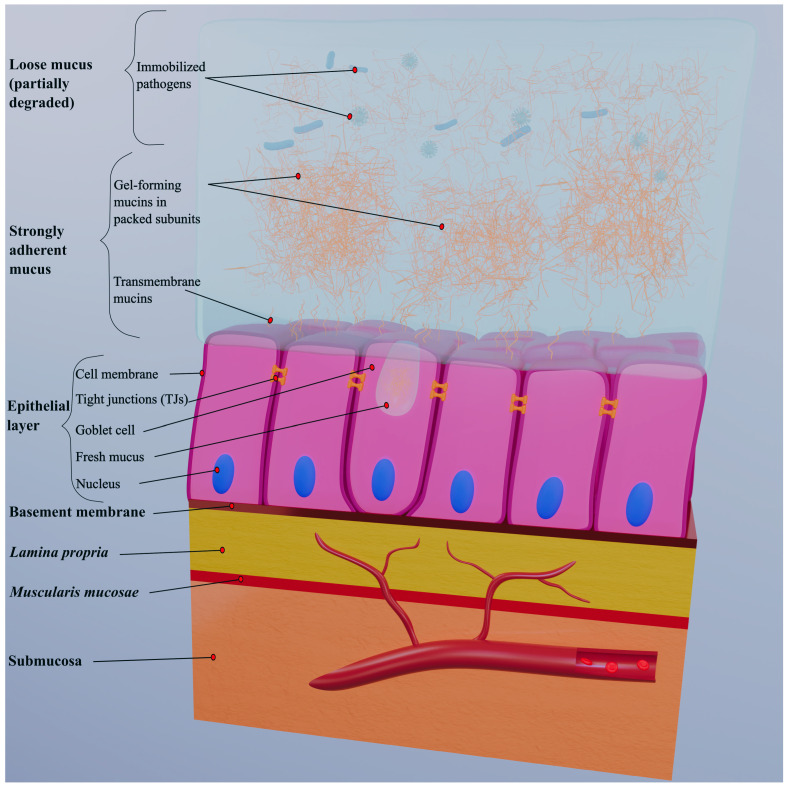
General scheme of mucosa and mucus.

Mucosa is defined as a moist tissue lining that covers all internal surfaces that are in contact with the external environment [15]. The structure of the mucosa varies depending on its location. Generally, mucosa consists of the outer epithelial layer covered with mucus, followed by vascularized lamina propria and muscularis mucosae (Figure 1). Mucus has multiple site-specific functions. For example, in the gastrointestinal tract, it provides lubrication and nutrient absorption; in the airways, it humidifies inhaled air; in the female genitourinary tract, it modulates permeability during the estrous cycle; and in the eyes, it reduces shear stress during blinking [16]. However, in all mucosae, it has one main function in common. This function is to protect underlying tissues from potentially harmful factors (e.g., dust, viruses, bacteria, toxins). Mucus is continuously secreted from the goblet cells or glands in the epithelium and forms a viscoelastic gel coating. The main component of mucus is water, which comprises up to 95% of its weight, whereas the other constituents include mucins (0.2–5%), lipids (1–2%), salts (0.5–1%), globular proteins (c.a. 0.5%), cells, DNA, and cellular debris [17].

As the second most prevalent part of mucus, mucins are glycoproteins with more than 50% of carbohydrates in their structure. There are 22 MUC mucins (e.g., MUC2) that are currently recognized and can be classified into three groups, i.e., membrane-bound mucins and secreted mucins, which can be further subdivided into gel-forming and nongel-forming (soluble) mucins [18]. Membrane-bound mucins (also transmembrane mucins) are cleaved at the stage of biosynthesis but remain attached to cellular membranes through strong noncovalent bonds [19,20]. Their role is not completely understood to date, but it is known that they participate in immunoregulation, form the glycocalyx of enterocytes in the intestines, and can be found in different parts of the organism outside the mucosae. On the other hand, gel-forming mucins are secreted from goblet cells or secretory glands and are characterized by high molecular weight, reaching up to 40 MDa [21]. In their structure, they possess cysteine-rich domains, which, through dimerization, form inter- and intramolecular disulfide bridges, which result in a mesh-like structure formed together with other components of mucus [18].

Mucus acts as the first line of defense in mucosae and filters out undesired molecules through three main protective mechanisms, i.e., steric, interactive, and dynamic [22]. Firstly, mucin mesh physically stops large molecules, preventing their diffusion and trapping them. Mesh pore sizes can vary between different mucosae but are usually within the 50–1000 nm range. Molecules smaller than 100–200 nm can effectively diffuse following Brownian motion. However, it was observed that molecules with sizes comparable to or larger than mucin mesh pores can also permeate through mucus, but only in a limited capacity. This is explained by the fact that mucus is a dynamic structure, and due to its continuous movement, the mesh spacing constantly changes, as explained in more depth in a recent review article [23]. Furthermore, mucins tend to form packed, mobile subunits that immobilize larger particles (>200 nm) attempting to pass through them, but the same particles might easily pass between them, as conceptualized by Menzel et al. [24]. Moving forward, the interactive filtering mechanism further hinders the transport of molecules based on their physicochemical surface properties. The majority of mucins contain proline, threonine, and serine (PTS) domains, which contain sialic and sulfonic acids, thereby endowing mucus with a negative charge [25,26]. This causes repulsion of negatively charged molecules and, in contrast, immobilization of positively charged molecules through electrostatic bonding. Similarly, hydrophobic cysteine domains in mucins interact with hydrophobic molecules and decrease their mobility. In summary, both steric and interactive barriers selectively choose which particles may reach the epithelium based on their aforementioned properties. On the other hand, the dynamic mechanism focuses on the removal of already trapped foreign particles through mucus turnover. The time required for mucus to be completely replaced varies among different mucosae and depends mainly on secretion rate, enzymatic and bacterial degradation, and mechanical forces, e.g., peristalsis in the gut, blinking in the eyes, and ciliary movement in the respiratory tract [16]. The exact turnover time is difficult to determine, and different data can be found throughout the literature, but it can be stated that the quickest removal occurs within minutes for ocular and respiratory mucus, followed by vaginal mucus turnover. The slowest mucus turnover can be found in the oral cavity, reaching up to 24 h, and in the gastrointestinal tract, even taking up to 48 h [8].

Steric, interactive, and dynamic protective mechanisms found in mucosae form an altogether difficult-to-bypass barrier. This poses a serious challenge to the delivery of therapeutics, especially those in classes II and IV of the Biopharmaceutics Classification System (BCS). However, that is not all. Mucus contains various enzymes, such as different proteases, depending on the mucosa location [27]. These enzymes cause degradation of therapeutics, thus increasing the amount of the drug necessary for a therapy. Furthermore, the situation becomes more complex if the mucosa is in a diseased state. Leal et al., in their review, described how the secretion and properties of mucus become different when affected by cystic fibrosis, chronic obstructive pulmonary disease, asthma, cancer, and dry eye syndrome [17]. Even though these are specific cases that affect a smaller group of patients, changes to mucus caused by localized disease should be taken into consideration when designing a drug delivery system because they may have an impact on its performance.

## 3. Mucointeractive Strategies

The defensive properties of mucus discussed in the previous section show that drug delivery systems capable of overcoming this barrier are required to effectively deliver therapeutics. Currently, there are three known types of desirable interactions between drug delivery systems and mucus (i.e., mucointeractions) that can be commonly found in the literature. One of them is mucoadhesion, which immobilizes a delivery system on the mucosa, thereby prolonging residence time and, to some extent, addressing the issue of the dynamic barrier (mucus turnover). Differently, mucodiffusive delivery systems aim to minimize interactions with the mucosa to improve the depth of penetration, thus countering the interactive barrier. On the other hand, mucolytic systems cleave the mucin mesh network, increasing pore sizes, which, in turn, increases permeation and counters the steric barrier. These mucointeractive strategies are known in the literature, and other good review articles are available on this topic, some of which are referenced here; thus, for this reason, they will be described only briefly.

**Figure 2:**
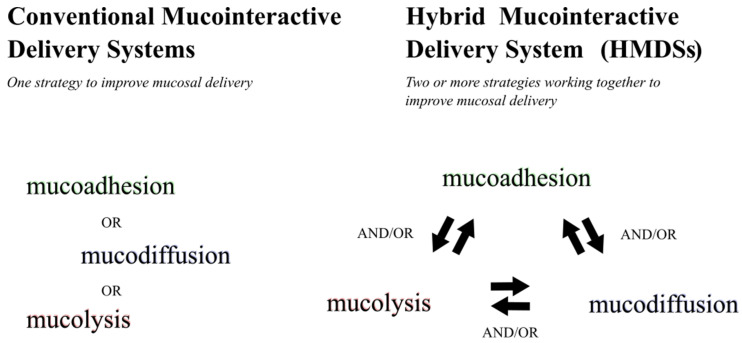
Scheme of conventional mucointeractive delivery systems vs. hybrid mucointeractive delivery systems (HMDSs).

It must be noted that the classification of these systems is not well established in the literature. For example, some authors separate mucointeractive strategies into mucoadhesion and mucopenetration, which are further subdivided into passive and active mucopenetrating systems, but the combination of mucoadhesion and mucopenetration is considered as a separate strategy [8]. Other authors, however, consider zeta potential-changing systems, which combine mucoadhesion and mucopenetration, still as mucopenetrating [9,25]. In this work, we assume a classification similar to what was proposed by Subramanian et al. [28] and separate basic mucointeractive strategies into mucoadhesion, mucodiffusion, and mucolysis, which are covered in this section. The systems that combine at least two of these basic mucointeractive strategies to further improve mucosal delivery are defined as hybrid mucointeractive delivery systems (HMDSs) and are discussed in Section 4 and Section 5 (Figure 2).

### 3.1. Mucoadhesion

**Figure 3:**
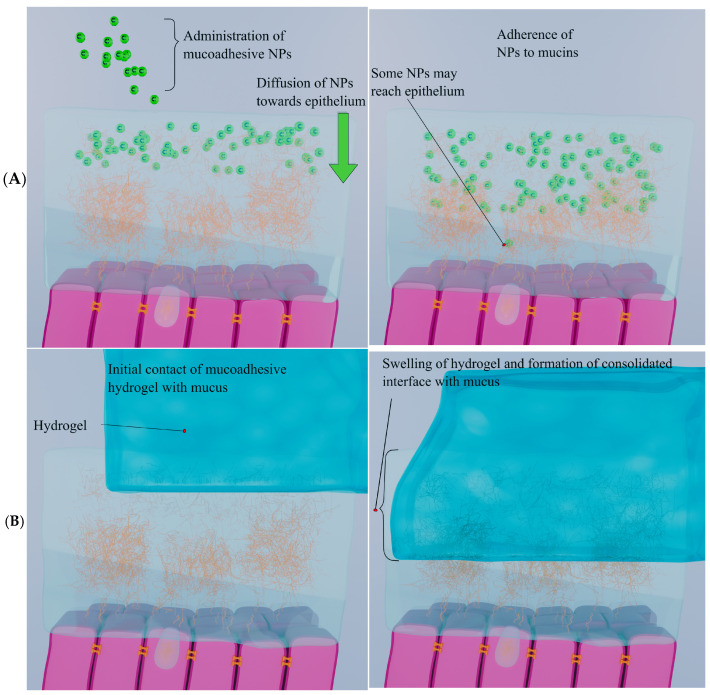
Mucoadhesive systems interacting with mucus: (**A**) upon administration, mucoadhesive nanoparticles spread across mucus, become immobilized through interactions with mucins, and provide prolonged drug release; (**B**) mucoadhesive macrostructures (e.g., hydrogel) undergo swelling, their polymeric chains interpenetrate and bond with mucins, forming a strong consolidated interface providing prolonged and localized release at the site of application.

Bioadhesion is defined as prolonged adherence of two materials facilitated by interfacial forces, where at least one is of biological origin [29]. If one of these materials is a mucosa covered with mucus, then such adherence is referred to as mucoadhesion (Figure 3). This type of adhesion is a complex phenomenon, and currently, there are six theories that together help to better understand this process: electrostatic theory, wetting theory, adsorption theory, diffusion theory, mechanical theory, and cohesive theory [30]. The mechanism of mucoadhesion can be subdivided into two stages [31,32]. At first, the contact stage takes place. The polymeric system swells and spreads on a mucosa (wetting theory). The surface roughness of the polymeric system and mucosa also plays a key role in this process (mechanical theory). This step is followed by electrical double layer formation (electrostatic theory), as well as initial formation of bonds at their interface, such as hydrogen bonds and Van der Waals forces (adsorption theory). Chemisorption may also occur due to the formation of ionic bonds and/or covalent bonds. The second stage is referred to as the consolidation stage. The polymeric system swells due to moisture, and the polymeric chains interpenetrate with mucus proteins (diffusion theory). At this point, the entangled polymer chains and proteins further form bonds with each other, and a strong consolidated interface is formed (cohesive theory). Although this is a general representation of the mucoadhesion process, it is important to note that the form of the system plays a crucial role. For example, the rheological properties of the liquid formulation will have a significant impact on mucoadhesion, whereas in the case of solid formulations, swelling properties will be one of the main factors affecting their adherence to the mucosa.

Many other polymer properties dictate its mucoadhesive abilities and should be taken into consideration, such as molecular weight, chain length, viscosity, degree of crosslinking, flexibility of polymeric chains, surface charge and degree of ionization, and water uptake, but its mucoadhesive properties will also be affected by the pH of the mucosa [33,34,35]. It can be seen that these properties are closely related to the mechanisms of mucoadhesion previously described, and they affect the polymer’s ability to swell, interpenetrate, and bond with the mucosa. However, considering the strength of mucoadhesion, adsorption theory (or chemisorption) is one of the most impactful, namely, bond formation between a polymer and mucosa. Based on this, mucoadhesive polymers can be subdivided into first-generation and second-generation materials [15]. The first generation of mucoadhesive materials includes cationic, anionic, and non-ionic polymers. Cationic polymers bond with negatively charged components of mucus (e.g., sialic acid) through electrostatic interactions due to the presence of amine groups that become protonated in acidic environments (below their pKa). The most popular and widely used polymer in the field of mucoadhesion is chitosan, which is a natural polymer with pKa ~6.3 obtained through deacetylation of chitin [36]. Although less researched, poly-L-lysine is another natural polycation that can be utilized in mucosal delivery [37]. Additionally, other polycations have also been explored for this purpose, such as poly(2-N,N-dimethylaminoethylmethacrylate) (PDMAEMA) [37], aminated cellulose [38], polyallylamine hydrochloride (PAH), and polyethylenimine (PEI) [38]. On the other hand, the majority of natural polysaccharides are negatively or neutrally charged; a wider range of such mucoadhesive polymers can be found in the literature. Some of such polyanions include alginate, pectin, xanthan gum, gum arabic, carrageenan, hyaluronic acid, and many others [39]. Anionic polymers interact with the mucosa mainly through hydrogen bonds and Van der Waals forces. However, similar to cationic polymers, their ability to form bonds with the mucosa is tightly linked to pH. At pH values above their pKa, the majority of the polymer’s ionizable groups are in their deprotonated form (e.g., –COO^−^), and negatively charged polymer chains repel negatively charged mucosa, whereas for pH values below pKa, these groups are mostly non-ionized (e.g., –COOH) and favor the formation of hydrogen bonds [40,41]. As a general rule of thumb, first-generation mucoadhesive materials can be organized based on their strength of mucoadhesion, starting with cationic polymers, followed by non-ionic polymers, and ending with the weakest anionic polymers due to their pH-dependent bonding/repulsion behavior.

Nevertheless, first-generation polymers form nonspecific bonds with various components of mucus, and thus, not all bonding sites fully contribute to the strength of mucoadhesion. For this reason, second-generation mucoadhesive materials are available as a viable alternative. These materials form covalent bonds with specific components of mucus due to the presence of functional moieties. The most well-known functional groups for this purpose are thiols, which were popularized in this field by Bernkop-Schnürch [42]. A plethora of different ligands and methods to introduce thiol groups into cationic, anionic, and non-ionic polymers have been presented in the literature. The mucoadhesive properties of thiolated polymers are attributed to the formation of disulfide bonds through reaction with thiol groups present in cysteine domains in mucins. However, thiomers are prone to oxidation, and thiol–thiol reactions may occur within the polymer itself before reacting with glycoproteins in mucus [43]. This can be prevented by introducing 2-mercaptopyridine or its analogs, resulting in so-called S-protected thiomers, which, besides improving stability, also improve their reactivity. For more information about thiolated polymers, the reader is referred to a very extensive review published a couple of years back by Leichner et al. [44]. Although thiols are well established in the literature and are the most commonly used, there are several other functional moieties that can serve a similar purpose. One such example is catechol-bearing ligands, which can bond covalently not only with thiols in cysteine but also with amine groups in mucins. However, catechols may also undergo undesired oxidation, resulting in the formation of quinone, but also possibly catechol-amine if the polymer backbone possesses amine groups like chitosan [45]. Nonetheless, several studies can be found on this topic and were partially covered in referenced works, but catechol-modified polymers are more popular in different research areas outside of mucoadhesion [46,47]. Although these two functional ligands are prone to oxidation and thus their stability is diminished, different, more stable options are also available. These include polymers modified with boranates [48], acrylates/methacrylates [49,50], maleimides [51], or N-hydroxysulfosuccinimide ester ligands [52], but very scarce data about the efficacy of these approaches is available as of now. These polymer functionalizations are discussed in more depth by Brannigan and Khutoryanskiy in their review [15].

Although second-generation materials have better mucoadhesive properties than noncovalent bonding polymers, too strong bonding might not necessarily be desired. If a polymer possesses too many ligands capable of bonding with mucins, its reactivity will be high, and it will become quickly immobilized at the upper layer of mucus, hindering further interpenetration [53]. Moreover, too strong mucoadhesion is not recommended for ophthalmic delivery systems, which might cause an increase in the viscosity of the tear film and result in eye irritation [52].

### 3.2. Mucodiffusion (Passive Mucopenetration)

**Figure 4:**
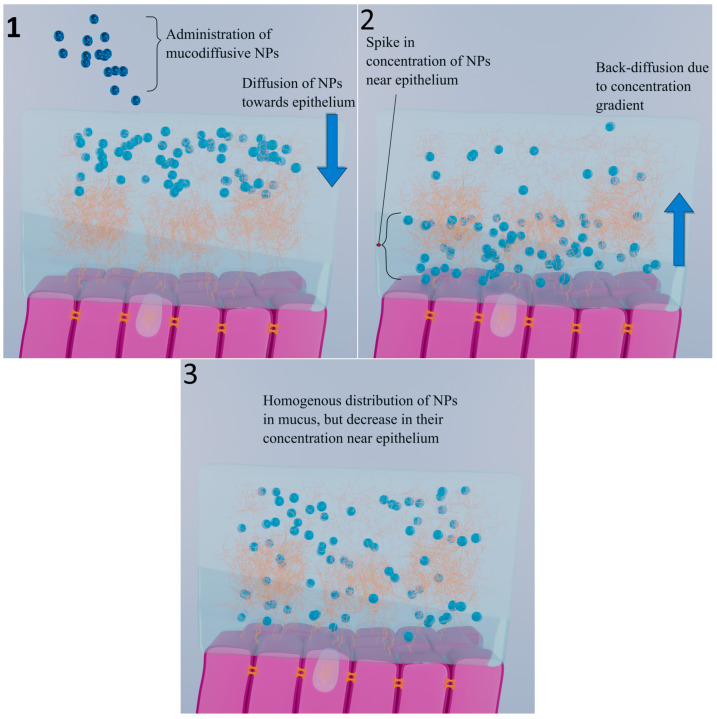
Mucodiffusive nanoparticles, upon administration, can easily diffuse through mucus. Initially, these nanoparticles diffuse between mucins towards the epithelium (**1**). Increasing concentrations of nanoparticles near the epithelium and their mucoinert properties result in their back diffusion towards the upper layer of mucus (**2**). This results in their homogeneous distribution throughout the mucus, but a small concentration near the epithelium negatively impacts the amount of nanoparticles that are taken up by the cells (**3**).

Mucodiffusion aims to minimize interactions with mucus to increase the depth of penetration of the delivery system (Figure 4). These systems can also be found in the literature under the names mucopenetrating or mucoinert. In contrast to mucoadhesive systems, which can be found in various forms, such as liquid formulations, solid systems, or particulate systems, mucodiffusion is only applicable to micro- or, preferably, nanocarriers from the perspective of drug delivery systems. The design of mucodiffusive particles must take into consideration the barrier properties of mucus. For this reason, these carriers must be small enough not to be immobilized by the steric barrier (interactions filtering), possess a negative or near-neutral surface charge to avoid bonding with anionic components of mucus, and be hydrophilic to avoid interactions with hydrophobic cysteine domains and lipids.

PEGylation refers to a modification of a molecule with poly(ethylene glycol) (PEG) [54]. In the 1970s, it was reported for the first time that such modification improved the circulation life of enzymes [55]. Not long after, PEGylation was also used to modify polymeric and liposomal nanocarriers, which decreased their plasma half-life and uptake by the reticuloendothelial system, improved their hydrophilicity, and decreased drug leakage from nanocarriers [56]. For these reasons, PEGylated nanocarriers are often referred to as stealth nanocarriers in the literature [57]. It was later noticed that these stealth properties could also be used in mucosal delivery to address challenges posed by the interactive barrier of mucus. There are two main methods that can be used to obtain PEGylated mucodiffusive nanocarriers: either through surface modification of already produced nanoparticles or through self-assembly with the use of nanoprecipitation or emulsification methods [58]. However, PEG moiety density on the nanocarrier surface, PEG moiety conformation, and molecular weight have a significant impact on the behavior of such nanocarriers in mucus. If nanocarriers are shielded with PEG of a high molecular weight, then their long chains will interact with and interpenetrate the mucus mesh, thus showing mucoadhesive properties [59]. Furthermore, if the density of the PEG moiety on the surface of a nanocarrier is low, then a mushroom conformation will be obtained, and the core material will not be fully shielded from interactions with mucus [60]. Thus, a high density of the PEG moiety on the surface of nanocarriers is required to cause steric hindrance of PEG chains and their outward stretching to obtain a brush conformation that minimizes interactions with mucus and improves mucodiffusion. However, a more recent study noted that an intermediate mushroom–brush conformation might be more desired, due to possible interactions between overly stretched PEG chains in brush conformations and mucins [61].

On the other hand, PEGylation is not the only approach to obtain the mucodiffusive properties of the drug carriers. A lot of articles discuss the examples of Norwalk virus and human papillomavirus, which can easily diffuse through mucus and serve as inspiration for the design of virus-mimicking particles [62]. These viruses possess a high density of evenly distributed positive and negative charges on their surface, which shields them from interactions with mucus. Particles that behave in a similar manner can be synthesized through the complexation of oppositely charged polyelectrolytes. Several examples include chitosan and chondroitin sulfate [63], chitosan and alginate [64], chitosan and hyaluronic acid [65], chitosan and poly(acrylic acid) [66], and poly(allylamine) and poly(acrylic acid) [67]. In contrast to polyelectrolyte complexes, virus-mimicking particles may be obtained with the use of polyzwitterions that possess cationic and anionic groups within their repeating units. However, so far, although only a few studies have tried to use these polymers as coatings to improve diffusion in mucus, the results are promising, and it was reported that such particles might promote transcellular transport without opening tight junctions [68,69].

Other, less popular approaches can be found in the literature [70]. One such example includes poly(2-alkyl-2-oxazoline) and its derivatives. It has been shown that grafting these polymers onto silica nanoparticles may provide similar or even better mucodiffusive properties than their PEGylated counterparts [71,72,73]. In addition, poly(vinyl alcohol) (PVA) was also considered as a possible alternative; however, the results were ambiguous. Popov et al. showed that mucodiffusion of nanoparticles can be improved with PVA if its degree of hydrolysis is sufficiently low [74]. On the contrary, Yang et al. showed that coating polystyrene particles with PVA of a different molecular weight did not improve diffusion in mucus and even decreased it, thus showing mucoadhesive properties [75]. Similar results were shown recently by Hu et al., who tested different coatings on poly(lactide-*co*-glycolide) (PLGA) nanoparticles and also concluded that PVA coating shows poor mucus diffusion [76]. Interestingly, in the same study, Pluronic F127, which is a copolymer of poly(ethylene oxide) and poly(propylene oxide), improved mucodiffusivity but was slightly surpassed by PEG and poly(dopamine) coatings in this regard. Poly(dopamine) coating was considered the best of all tested coatings, as it also improved cellular uptake.

Additionally, it is imperative to note that not only do the size and surface properties matter, but also the shape. Yu et al. showed that mesoporous silica in the form of nanorods had better mucodiffusion than nanospheres due to their possible rotational motion in mucus [77]. Similarly, Bao et al. tested short nanotubes, crosslinked short nanotubes, small nanospheres, large nanospheres, and long nanotubes prepared from α-lactalbumin [78]. They concluded that short nanotubes had the best mobility in mucus and exhibited the highest cellular uptake. Moreover, less rigid particles were reported to be more favorable than their crosslinked counterparts.

The topic of mucodiffusive particles was covered by others in several review articles [8,11,25,58,70].

### 3.3. Mucolysis (Active Mucopenetration)

**Figure 5:**
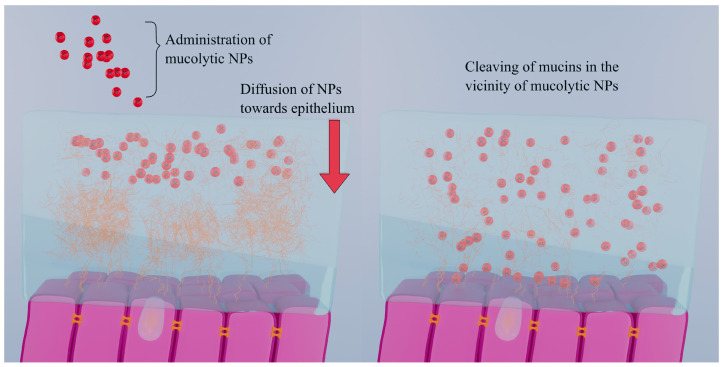
Mucolytic nanoparticles cleave mucins in their vicinity, which increases spacing within the mucin mesh. This allows these nanoparticles to diffuse deeper into the mucus and towards the epithelium. However, a disrupted mucin mesh may not provide sufficient protection from harmful pathogens until mucus is restored.

Of all three mucointeractive strategies, mucolysis is the newest one, and it aims to increase the depth of penetration similarly to mucodiffusion. Although this approach increases permeation similarly to other permeation enhancers, the majority of standalone permeation enhancers known in the literature affect tight junctions and epithelial cells to improve paracellular or transcellular transport of therapeutics [79]. In contrast, the mucolytic strategy focuses mainly on decreasing the viscosity of mucus and increasing the spacing between mucins for easier transportation of therapeutics towards the epithelium (Figure 5). There are several mucolytic agents that can be incorporated into delivery systems for this purpose, such as proteolytic enzymes (e.g., bromelain, papain, pepsin, trypsin), but also other molecules that break down disulfide bonds (e.g., N-acetylcysteine) [8,24]. In recent years, this strategy has been gaining more attention, and as of now, several approaches to obtain mucolytic delivery systems can be distinguished.

The most straightforward approach includes encapsulation of the mucolytic agent inside the carrier, which continuously releases mucus-cleaving substances once the system comes into contact with mucus. Unlike other kinds of mucopenetrating systems, this method is applicable not only to nanoparticulate carriers [80] but may also be used in macrostructures, e.g., hydrogels [81]. However, very little data is available about such systems as of now.

Particulate carriers can also be modified with proteolytic enzymes to form so-called “enzyme-decorated” nanoparticles. Here, the enzyme is attached to the polymeric carrier. These particles may be obtained by either physically attaching enzymes to oppositely charged polymers [82] or through covalent bonding between the two [83]. It must be noted that covalently bound enzymes were found to show better activity than physically attached enzymes [24]. Similarly, physical or covalent attachment of mucolytic agents may also be used for self-emulsifying drug delivery systems [84,85].

A special case of thiomers must be highlighted. As mentioned before, these thiol-bearing polymers bind with thiol groups in mucins, exhibiting mucoadhesive properties. However, due to the thiol–disulfide exchange reaction, they also possess mucolytic properties, similar to the mucolytic agent N-acetylcysteine [86]. A recent study showed that thiolated polyglycerol-based nanoparticles exhibit stronger mucolytic properties than a standalone N-acetylcysteine [87]. In another study, a comparison between papain- and cysteine-modified polyacrylic acid nanoparticles was conducted [88]. Cysteine-modified nanoparticles showed worse permeation than their non-modified counterparts. This might indicate that the mucoadhesive properties of thiolated polymers dominate their mucolytic properties.

The topic of mucolytic systems was discussed in more depth in a different article by Menzel and Bernkop-Schnürch [24]. However, since the mucolytic strategy disrupts the natural protective barrier of the mucosa, there may be some concerns about possible dangers. In their review article, these authors included a very broad discussion about safety and concluded that there are no points at which this approach is unsafe. On the other hand, a different point of view was presented by Chater et al. [89]. These authors reference a study that treated rat jejunum with N-acetylcysteine and observed acute mucosal damage [90]. Although in this case the damage was healed after 2 h, lack of protection even for a short duration might be potentially dangerous. Furthermore, one of the possible drawbacks of this method is that the delivered therapeutic is in direct contact with a mucolytic agent. For example, it is well known that proteolytic enzymes in the gastrointestinal tract are responsible for insulin degradation [91,92]. Obviously, a degradation of the therapeutic inside a mucolytic carrier containing such enzymes would be less than desirable; therefore, their compatibility should be taken into consideration. However, no study so far has shown whether such a phenomenon may or may not occur.

## 4. Particulate Two-in-One Hybrid Mucointeractive Delivery Systems

Nano- and microparticulate systems currently dominate the mucosal delivery research field due to several advantages, e.g., ease of administration, small size, protection of therapeutics, and broad distribution across the mucosa. The previously described mucointeractive strategies improve the delivery of biomolecules, which was confirmed by clinical studies, and many products are readily available on the market. However, in many cases, using a single mucointeractive strategy in a delivery system does not provide satisfactory delivery. Mucoadhesive NPs become entrapped in mucus and are removed before releasing therapeutics, which severely limits their efficacy, especially at fast-clearing mucosae. On the other hand, mucodiffusive NPs can diffuse through mucus fairly easily, but their mucoinert properties and changes in NP concentration gradients result in back diffusion, lowering the amount of NPs near the epithelium (Figure 4). It is also known that the epithelium favors positively charged particles for cellular internalization; thus, mucodiffusive NPs show generally lower cellular uptake than mucoadhesive NPs [93]. In recent years, nano- and microparticulate two-in-one HMDSs have emerged as a possible solution to overcome these challenges.

### 4.1. Zeta Potential-Changing Nanoparticles

**Figure 6:**
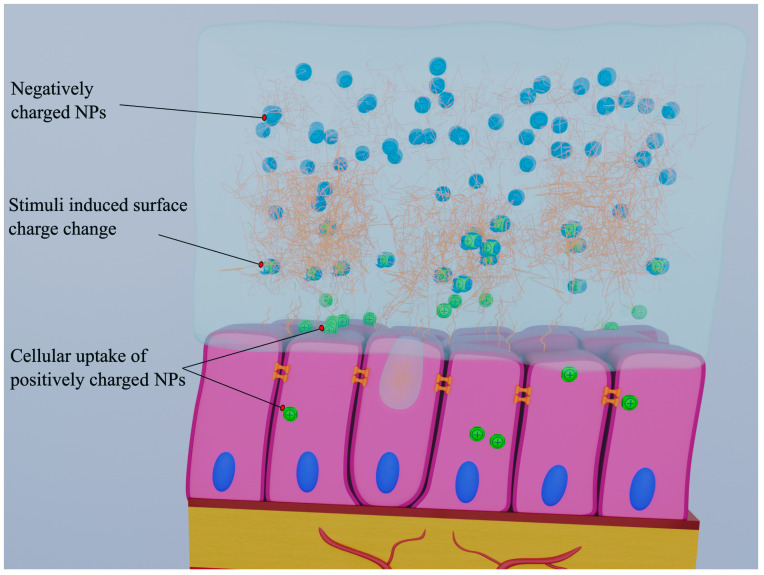
Zeta potential-changing systems are initially negatively charged and can easily move through mucus, behaving like mucodiffusive nanocarriers. Upon coming into contact with specific stimuli, their surface charge changes to a positive value, and these nanocarriers become mucoadhesive. The positive charge also promotes their cellular uptake and transport towards deeper layers of the mucosa.

Zeta potential-changing systems can be classified as HMDSs and are capable of switching their surface charge when specific stimuli are applied. A shift from negative to positive charge causes, subsequently, a change in how these particles interact with mucus, namely, they change from mucodiffusive to mucoadhesive (Figure 6). The proof of concept was first reported in 2015. Bonengel et al. produced zeta potential-changing particles by the synthesis of Polyethyleneimine-6-phosphogluconic acid, which was used in the formation of poly-ion complexes with carboxymethyl cellulose (CMC) [94]. The obtained particles initially had a negative zeta potential (−6 mV), but after coming into contact with intestinal alkaline phosphatase (IAP), the 6-PGA was cleaved from these particles. This changed their zeta potential to a positive value (+3 mV). Perera et al. produced particles via polyelectrolyte complexation of separately modified CMC and chitosan with phosphotyrosine [95]. The zeta potential increased from an initial value of −8 mV to as much as +8 mV after elimination of phosphotyrosine with IAP. A later study confirmed that such systems are, in fact, capable of switching their profile from mucodiffusion to mucoadhesion due to a change in their surface charge [96]. Phosphotyrosine (Ptyr) was covalently attached to PEG diamine to obtain mucodiffusive particles. Enzymatic degradation of Ptyr decreased the diffusion of particles through mucus and, at the same time, increased the dynamic viscosity of polymer/mucus mixtures, indicating mucoadhesive properties.

Since this concept was reported, more zeta potential-changing systems have been developed for mucosal delivery. Currently, several different stimuli are recognized in the literature that can induce surface charge change in nanocarriers, i.e., enzymatic, pH-triggered, redox, and exogenous stimuli [97]. Enzyme-induced hydrolysis causes the removal of a negatively charged outer layer, exposing a positively charged inner layer of the nanocarrier. The previously described systems in this section utilized alkaline phosphatase and changed their charge from negative to positive in its presence. The use of this particular enzyme is dictated by its high prevalence in humans and animals. There are three tissue-specific isozymes, one of which is the IAP present in the gastrointestinal tract [98]. The tissue-nonspecific isozyme of alkaline phosphatase can be found in the vagina [99], lungs [100], nasal cavity [101] and eyes [102]. Thus, such systems that change their surface charge due to alkaline phosphatase can be applicable to different mucosae. Despite this, reports on the use of different cleaving enzymes can also be found, e.g., heparanase [103].

The physiological pH in the majority of healthy tissues is slightly basic at around 7.2. On the other hand, the pH of mucus differs from this value and is usually more acidic, i.e., nasal cavity and lungs (pH ~5.5–6.5), eyes (pH ~7.8), oral cavity (pH ~6.2–7.4), premenopausal vagina (pH ~4.0–5.0), and gastrointestinal tract (pH ~1.0–7.0) [104]. This fact can be used to induce surface charge changes from negative to positive in nanocarriers that possess protonable functional groups on their surface or a negatively charged coating soluble in a specific pH range. Naeem et al. synthesized polyethylenimine lipid nanoparticles coated with Eudragit^®^ that could maintain a negative charge throughout the GI and switch to positive in the colon due to pH-triggered removal of the coating [105]. In addition, Xi et al. produced NPs based on PLGA-PEG with charge-changing properties that promoted diffusion through mucus and enhanced epithelial transcytosis [106]. In a similar study, pH-responsive PEGylated PLGA NPs were capable of changing their zeta potential based on the pH of the medium [107]. It was shown that the initial potential of −2 mV increased to +14, +22, and +27 mV for pH of 6.2, 5.0, and 4.0, respectively. Although pH-triggered zeta potential-changing systems are promising, the pH of mucus can be affected by multiple factors. This must be taken into consideration when designing such a system for a particular treatment.

Both redox and exogenous (i.e., light, heat) stimuli were proven to be capable of causing surface charge change that promotes cellular uptake, mainly for imaging or cancer therapy applications, but to the best of our knowledge, no studies specifically on mucosal delivery have been presented so far. Polymeric stimuli-triggered zeta potential-changing systems were recently reviewed elsewhere and will not be described here in more detail [97,108].

### 4.2. Zeta Potential-Changing Self-Emulsifying Drug Delivery Systems

Possible alternatives to the presented polymeric nanoparticles are self-emulsifying drug delivery systems (SEDDSs). SEDDSs are defined as isotropic mixtures of oils, solid or liquid surfactants, or, alternatively, one or more hydrophilic solvents and cosolvents/surfactants [109]. Contrary to polymeric particles, SEDDSs are initially liquid and form oil/water nanoemulsions once introduced into the aquatic environment (e.g., physiological). These systems were designed mainly for the delivery of poorly soluble or water-insoluble therapeutics, such as BCS Class II and IV drugs, and have been in use for over 20 years. The oil component serves mainly as a solvent for a lipophilic drug, whereas the surfactant plays a key role in emulsification and should possess a high hydrophilic–lipophilic balance and hydrophilic properties. Similarly to what was discussed previously, SEDDSs can also possess zeta potential-changing properties, which make them even more promising from a transmucosal delivery standpoint. For example, Griesser et al. produced SEDDSs with phosphorylated starch and phosphorylated hydroxypropyl starch using surfactants and cosolvents [110]. In the presence of IAP, phosphate was released, and zeta potential shifted from −6.3 mV to +1.0 mV. Moreover, pretreatment with IAP caused worse diffusion through mucus in Transwell chambers compared to untreated SEDDSs, indicating a change in mucointeractive properties. Other systems similar to this one have been presented in the literature. As mentioned in the case of polymeric zeta potential-changing particles, several stimuli can cause a shift in surface charge, but to the best of our knowledge, only enzymatic stimuli have been used in SEDDSs for this purpose so far. Despite their many advantages, SEDDSs have some drawbacks, e.g., chemical instability of drugs, precipitation of lipophilic drugs in the presence of a volatile cosolvent in a gelatin capsule, and disrupted homeostasis of the mucosa due to high amounts of surfactants [111]. These zeta potential-changing SEDDSs were very recently reviewed by Arshad et al. [112].

### 4.3. Mucoadhesion-to-Mucodiffusion Systems

Although the zeta potential-changing strategy was shown to promote the cellular uptake of positively charged particles, it may not provide sufficiently prolonged residence time and localized delivery due to initially mucodiffusive properties that first must come into contact with specific stimuli to undergo a charge change. This issue was partially addressed by a group that proposed a reversed approach and encapsulated mucodiffusive PEGylated lipids in mucoadhesive alginate beads [113]. However, the obtained results did not show extended retention of encapsulated vesicles. The authors suggested that a carrier with stronger mucoadhesive properties, e.g., a cationic or thiolated polymer, could potentially provide the desired effect. Such a system, consisting of mucodiffusive α-lactalbumin peptosomes encapsulated in thiolated microspheres, was designed by Zhao et al. [114]. Rectal administration of microspheres assessed on a mouse model showed that thiolated carriers could last up to 6 h in the colon compared to 1 h for non-encapsulated peptosomes. In addition, oral administration showed that standalone peptosomes could reach the colon in 2 h and were excreted after 4 h, whereas thiolated microspheres took 12 h to reach the colon and were still present after 18 h. Moreover, studies confirmed mucoadhesive and mucodiffusive properties of this system. This mucoadhesion-to-mucodiffusion approach was also verified by other authors. Li et al. loaded poorly water-soluble and hydrophobic quercetin into positively charged micelle-like nanoparticles and encapsulated them into oxidized starch microgels [115]. These microgels provided protection from gastric enzymes, and due to their mucoadhesive and pH-responsive properties, prolonged and sustained release of nanoparticles was accomplished. Furthermore, encapsulated nanoparticles showed two times better intestinal coverage after 1 h of administration than standalone nanoparticles. More recently, mucodiffusive nanotubes (α-lactalbumin) were introduced into low-methoxy pectin microgels [116]. In this case, mucoadhesive microcarriers also increased retention and showed controlled release of nanotubes in the intestine, even for up to 12 h.

**Figure 7:**
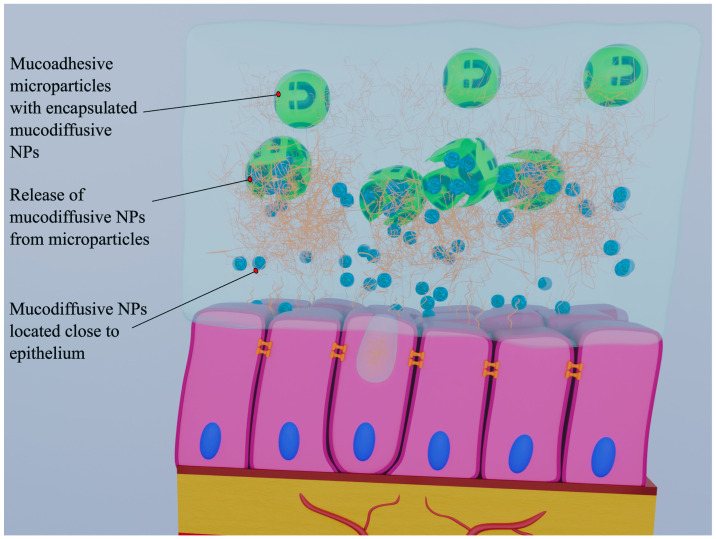
Mucoadhesion-to-mucodiffusion systems, upon administration, become immobilized in mucus and release mucodiffusive nanoparticles. These nanoparticles easily diffuse throughout the mucus anad towards the epithelium. The presence of a mucoadhesive component in this system not only ensures localized release but may also possibly prevent back diffusion of mucodiffusive nanocarriers by contracting the mucin mesh.

Three out of the four presented systems showed the desired effect and improved retention of mucodiffusive nanocarriers by the epithelium, but only in one of these cases did nanocarriers possess the positive surface charge desired for high cellular uptake. We speculate that the mechanism behind it is closely linked to the mucoadhesive properties of microcarriers. It is a known fact that mucoadhesive particles can cause contraction of mucin gel, resulting in the formation of larger pores, which allow for some of the particles to diffuse deeper [117]. This may potentially result in the release of drug-loaded nanocarriers closer to the epithelium, while contracted mucin mesh partially prevents their back diffusion, ensuring a high concentration of NPs near the epithelium (Figure 7). However, further studies are required to prove or disprove such a hypothesis for these systems.

### 4.4. Mucolytic–Mucoadhesive Systems

The combination of mucodiffusion and mucoadhesion is not the only approach that is available to achieve hybrid delivery. Although the underlying mechanism is different, it can be considered that mucodiffusion is closely related to mucolysis, since they both aim at improving the traversal of encapsulated biomolecules through mucus. For this reason, one can be substituted by the other to obtain a hybrid system with mucolysis and mucoadhesion. This synergy similarly addresses the issue of mucoadhesive nanoparticles that quickly adhere to the surface of mucus, which is followed by too early drug release and its removal with mucus clearance. Mucolysis could increase the depth of diffusion by cleaving the mucus structure, whilst mucoadhesion could prevent back diffusion and prolong release closer to or even past the epithelium. This was partially confirmed by Köllner et al., who synthesized thiolated PAA (PAA-cys) and papain-modified PAA (PAA-pap) conjugates and, through ionic gelation, produced nanoparticles [88]. It was shown that PAA-cys-pap exhibited 2.0-fold higher diffusion in mucus than PAA-cys nanoparticles without enzymes, whereas the mucoadhesion was 1.9-fold higher in comparison to PAA-pap nanoparticles. Similarly, Zafar et al. obtained polycarbophil (PCP) nanoparticles modified with thiol groups (PCP-cys) and also with papain (PCP-cys-pap) [118]. PCP-cys-pap showed better mucus penetration than PCP-cys, but worse than PCP-pap, in a silicone tube diffusion test. Furthermore, mucoadhesion was increased two times for thiol-modified particles in comparison to their counterpart. In a different approach, besides the thiolated copolymer (thiolated hyaluronic acid–co-oleic acid) and papain enzyme, a targeting moiety for *H. Pylori* bacteria (urea) was also introduced into the system [119]. As expected, the presence of papain increased the depth of penetration, whilst the thiolated copolymer increased the residence time. The addition of urea improved the growth inhibition properties of the system *in vitro* and provided a ca. 30-fold better reduction in bacteria than other tested formulations *in vivo*. This study proved that targeting moieties can, in fact, be successfully combined with hybrid mucointeractive strategies to obtain even better synergy and thus treatment.

**Figure 8:**
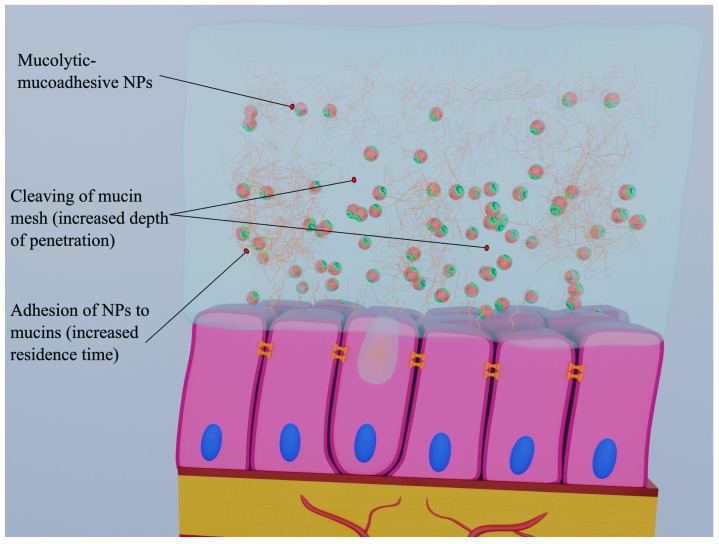
Mucolytic–mucoadhesive systems utilize both mucointeractive strategies at the same time. Mucolytic properties allow nanocarriers to diffuse deeper into mucus, whereas mucoadhesive properties immobilize nanocarriers, increasing residence time close to the epithelium.

It must be noted that these particular two-in-one HMDSs differ significantly from the other cases discussed in this section so far. Here, the change from one mucointeractive strategy to another does not occur, but both mucoadhesion and mucolysis act simultaneously (Figure 8).

## 5. Particles-in-Macrostructure Hybrid Mucointeractive Delivery Systems

Incorporation of nanocarriers into macrostructures like gels, sponges, films, and foams has been used in drug delivery for several decades [120,121]. These structures protect therapeutics from degradation, minimize local toxicity, and provide better control over release [122]. In these types of hybrid systems, the role of the macrostructure is to provide prolonged contact with the mucosa, as well as sustained and localized release of nanocarriers. For this purpose, first- and second-generation mucoadhesive polymers can be chosen. Regarding the nanocarrier, polymeric nanoparticles can be easily modified to obtain desired mucointeractive properties; however, their cargo release mechanism is usually based on water-driven diffusion, osmotic pumping, and erosion [123]. This partially limits their use in hydrated macroscale systems like hydrogels. On the other hand, lipid nanocarriers, e.g., liposomes, niosomes, solid lipid nanoparticles, nanostructured lipid carriers, can maintain their cargo in water, making them more promising from this standpoint [124]. However, similarly to polymers, lipid nanoparticles are also affected by mucus and face issues regarding their diffusion [125]. Surface modifications must be considered to obtain the desired mucointeractive properties, which can be done by coating lipid nanocarriers with different polymers [126]. Moreover, polymer coatings were shown to improve the long-term stability of liposomes [127].

**Figure 9:**
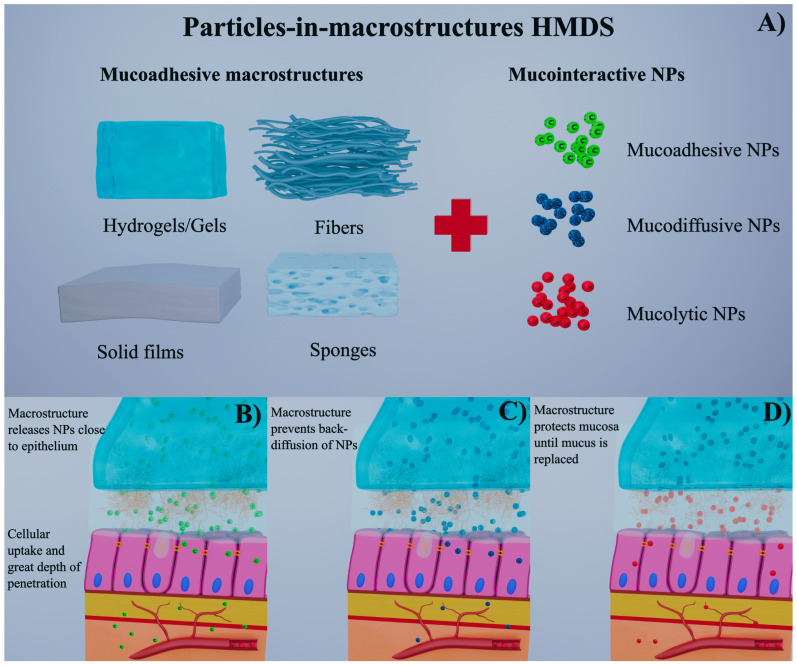
(**A**) Particles-in-macrostructure HMDSs comprise two components: a mucoadhesive macrostructure (e.g., hydrogels/gels, fibers, solid films, sponges) and mucointeractive nanoparticles. (**B**) The macrostructure not only provides prolonged residence time on the mucosa but also releases nanoparticles close to the epithelium, preventing positively charged nanoparticles from being immobilized in the upper layer of mucus. (**C**) The macrostructure may prevent back diffusion of nanoparticles, ensuring their high concentration near the epithelium. (**D**) The macrostructure may act as an additional protection from pathogens for the mucosa until new mucus replaces old mucus disrupted by mucolytic nanoparticles.

This section discusses both mucointeractive lipid-based and polymeric nanocarriers, which were combined with mucoadhesive macrostructures, resulting in HMDSs (Figure 9).

### 5.1. Mucoadhesive Nanocarriers

One of the possibilities includes the incorporation of mucoadhesive nanocarriers into a mucoadhesive macrostructure. The emphasis on the mucoadhesive properties of nanocarriers may be less significant than in the other cases discussed in this work, as it is already provided by mucoadhesive macrostructures themselves. Here, the positive charge of nanocarriers plays a more crucial role in promoting cellular uptake. Several authors presented valuable insight into the efficacy of such an approach. Multiple reports are available on mucoadhesive nanocarriers in mucoadhesive macrostructures and are shown in Table 1. However, incorporation of such nanocarriers into a macrostructure can have a significant effect on their performance.

Recently, a system consisting of chitosan NPs with insulin incorporated into hydroxypropyl methylcellulose (HPMC)-based films was evaluated [128]. It was reported that standalone chitosan NPs showed quicker release *in vitro* in comparison to NP-loaded films. The release kinetics of chitosan NPs did not change upon loading into the film and followed the polymer swelling and erosion mechanism. The hybrid system caused a smaller decrease in blood glucose concentration *in vivo* than subcutaneously delivered insulin. However, the decrease was continuous even after 5 h for the hybrid system, whereas the subcutaneously delivered insulin started showing an increase in glucose levels after 3 h. This proves the prolonged and sustained release properties of this particular HMDS. However, chitosan NPs decreased the mucoadhesive strength of HPMC-based films from 3.1 N to 2.3 N, which was attributed to the interruption of the continuous film structure by NPs, as explained by the authors.

Silvestre et al. aimed at improving the buccal delivery of water-insoluble curcumin through its encapsulation in chitosan/sodium alginate NPs, which were then incorporated into sodium alginate films [132]. Here, loading chitosan/sodium alginate NPs into sodium alginate films showed an insignificantly small increase in mucoadhesive force. The standalone NPs released less curcumin than the same NPs loaded into sodium alginate films, possibly due to the association between curcumin and the polymer network of the film. Moreover, the incorporation of NPs into films did not change their release kinetics, but in this case, the release mechanism was different from that of the previously mentioned system, which released insulin and followed non-Fickian diffusion.

On the other hand, Mazzarino et al. encapsulated curcumin in chitosan-coated PCL NPs and loaded them into chitosan films. They observed that chitosan–PCL NPs in films released 3 times more poorly soluble curcumin *in vitro* than standalone chitosan films [131]. It was also highlighted that the high-molecular-weight chitosan in films showed a slower release rate of NPs in comparison to medium-molecular-weight chitosan. Furthermore, chitosan–PCL NPs decreased the swelling capacity of chitosan films, and since swelling has a direct effect on mucoadhesion, this property could be negatively affected. However, in this study, mucoadhesion tests were not included.

A similar effect of NPs on the swelling properties of films was reported in another study [129]. Here, chitosan NPs in HPMC/poly(acrylic acid) films increased the initial water uptake during the first 30 min, but after 60 min, the swelling index decreased for all NP-loaded films, but not for films without NPs. This was attributed to the formation of hydrogen bonds between NPs and the film. This data was correlated with mucoadhesive properties, and as expected, films without NPs showed the highest detachment force needed to separate them from the mucosa. The *in vitro* release from NPs in the film was not significantly different in comparison to the release from the film itself.

Lipid-based nanocarriers possess one crucial advantage over polymeric NPs and can be incorporated into mucoadhesive gels, which are easily applicable, especially if they possess in situ gelling properties. One such HMDS was prepared by coating curcumin-carrying lipid-core nanocapsules (LNCs) with chitosan and loading them into an HPMC/PEG-PPG copolymer gel [135]. A washability test on porcine oral mucosa of standalone chitosan-coated and uncoated LNCs did not show any significant differences, and complete removal of nanocarriers was observed after 90 min in both cases. In contrast, nanocarrier-loaded gels provided sustained release of LNCs for the entire test duration (up to 8 h); moreover, chitosan-coated LNCs released from the gel showed the best permeability through the mucosa.

In a different study, chitosan-coated liposomes were incorporated into a poly(acrylic acid) gel, and the effect of pH on drug release was evaluated [137]. After 24 h, uncoated liposomes in the gel released 72% of the drug at pH 7, whereas chitosan-coated liposomes in the gel released only 49%. A decrease in pH to 6 caused protonation of chitosan amine groups, resulting in 75% drug release, similar to uncoated liposomes in the gel at this pH. For this reason, the pH of the targeted mucosa must be taken into consideration when using chitosan as a coating of nanocarriers. Nonetheless, the release from coated and uncoated liposomes in the gel was overall lower than for the drug loaded directly into the standalone gel, which showed up to 86% release after 24 h at both tested pH levels.

**Figure 10:**
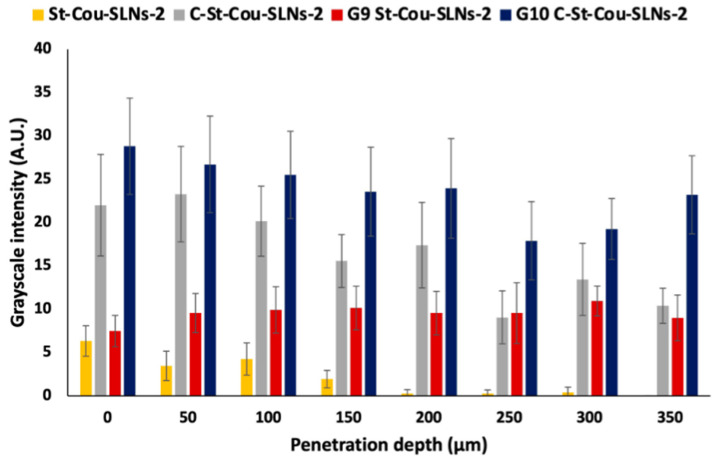
Penetration depth of drug delivery systems through bovine urinary bladder mucosa; (St-Cou-SLNs-2)—standalone nanocarriers, (C-St-Cou-SLNs-2)—standalone chitosan-coated nanocarriers, (G9 St-Cou-SLNs-2)—nanocarriers in PEG-PPG copolymer/poly(acrylic acid) gel, (G10 C-St-Cou-SLNs-2)—chitosan-coated nanocarriers in PEG-PPG copolymer/chitosan gel [138], and CC BY.

One of the studies must be clearly highlighted as it provides detailed insight into the efficacy of mucoadhesive nanocarriers in the mucoadhesive macrostructure approach. Shawky et al. aimed to deliver quercetin for the treatment of bladder cancer by producing solid lipid nanoparticles (SLNs) and loading them into in situ gelling mucoadhesive gels [138]. Positively charged chitosan-coated SLNs were incorporated into PEG-PPG copolymer/chitosan gels, whereas negatively charged uncoated SLNs were incorporated into PEG-PPG copolymer/poly(acrylic acid) gels. Both systems showed good mucoadhesion, and the presence of SLNs had no significant effect on gel strength and its viscosity. Similarly to what was observed in other studies, loading uncoated SLNs into gels retarded drug release *in vitro,* and standalone SLNs released 30% and 90% in comparison to uncoated SLNs-in-gel, which released 18% and 70% after 22 h and 92 h, respectively. It must be noted that after 142 h, the amount of released drug was comparable in both cases. Chitosan-coated SLNs-in-gel presented a slower release profile than their counterparts. Moreover, a washability test showed that 41% of chitosan-coated SLNs were retained on the bladder mucosa, whereas only 7% of uncoated SLNs remained. Although this result differs from the previously discussed study [137], similarly, here, the incorporation of uncoated and coated SLNs into gels improved retention on the mucosa, resulting in 53% and 65.5% of formulations retained at the end of the test, respectively. Most importantly, the depth of diffusion through the bladder mucosa was evaluated (Figure 10). Uncoated SLNs showed the lowest depth of penetration and were not detected past 150 μm. Loading them into gels improved their depth of penetration, and fluorescent intensity was fairly constant up to 350 μm. On the other hand, coating SLNs with chitosan allowed for greater penetration, but the intensity decreased significantly with depth. The best penetration was obtained in the case that combined the mucoadhesive gel and chitosan-coated SLNs. Such a finding demonstrates the viability of mucoadhesive nanocarriers in a mucoadhesive macrostructure approach. Moreover, this system offers great control over the depth of diffusion and can be adjusted for a particular treatment.

### 5.2. Mucodiffusive Nanocarriers

The inertness of mucodiffusive nanocarriers towards mucus is, at the same time, their advantage and disadvantage. Combining them with mucoadhesive macrostructures could potentially prevent their back diffusion while maintaining their great mobility in mucus close to the epithelium. So far, such nanocarriers have been incorporated into various mucoadhesive macrostructures in the form of sponges, foams, hydrogels, films, and electrospun fibers (Table 2).

PEGylation is the most common method to provide nanocarriers with mucodiffusive properties, as highlighted previously in Section 3.2. However, in the case of lipid-based nanocarriers, PEGylation is also frequently used as a way to improve their stability and limit aggregation. For this reason, only research articles in which authors highlighted possible mucodiffusive properties of PEG coating at any point in their work were chosen and presented in this part.

Wu et al. studied commercially available transfection agents of nucleic acids for vaginal delivery and found that they were taken up only by cell debris during the estrous cycle, thus not reaching the target tissue [139]. Pretreatment of the vaginal cavity with citric acid showed slight but unsatisfactory improvement. To improve delivery, they designed an HMDS composed of PEGylated lipoplexes loaded into freeze-dried alginate sponges. Such an approach provided a 6-fold higher uptake in vaginal tissue in comparison to conventional standalone cationic lipoplexes. A similar system was proposed by other authors, who produced hydroxyethyl cellulose hydrogels loaded with lipoplexes, and different PEG coatings were evaluated [142]. Lipoplexes coated with 2000 Da PEG proved to minimize interaction with mucus to a greater extent than 750 Da PEG, and their incorporation into a hydrogel did not affect its mucoadhesive and mechanical properties significantly.

Deformable liposomes can be considered as an alternative to conventional liposomes. They were proposed for the first time in the 90s, and due to their smaller Young’s modulus, they can easily deform, potentially improving their permeation in transdermal or transmucosal delivery applications [151]. A comparison of deformable PEG-liposomes, PEG-liposomes, and conventional liposomes, which were loaded into chitosan hydrogels, was recently conducted [143]. Although the fastest release *in vitro* was obtained for standalone chitosan hydrogels, hybrid systems with deformable PEG-liposomes showed the second fastest release, followed by PEG-liposomes in hydrogels and conventional liposomes in hydrogels; thus, in this case, higher deformability allowed for quicker release. Neither of the liposomes affected the rheological properties of the hydrogels, but both deformable PEG-liposomes and PEG-liposomes increased mucoadhesion. On the other hand, Vanić et al. prepared a similar system composed of deformable PEG-liposomes and Poly(acrylic acid) hydrogels [140]. In their study, they loaded nanocarriers separately with two types of drugs (i.e., hydrophilic and lipophilic) for comparison. Similarly, deformable PEG-liposomes incorporated into hydrogels showed slightly quicker release *in vitro* than conventional liposomes in hydrogels for both drug types, and the quickest release profile was obtained for standalone hydrogels. However, both deformable PEG-liposomes in hydrogels and conventional liposomes in hydrogels showed faster release of hydrophilic drugs than lipophilic drugs within 24 h. In the case of standalone hydrogels, without the use of nanocarriers, the release was not affected by the drug type. Using deformable PEG-liposomes instead of PEG-liposomes might be considered as a viable alternative if loading nanocarriers into macrostructure slows down the release too much, but their permeability as an HMDS must be further evaluated.

Unlike most of the HMDSs discussed here, two systems for delivering water-soluble therapeutics were designed. One such system, composed of PEG-liposomes in a mucoadhesive buccal film, was used to deliver vitamin B6, which, despite great water solubility, has poor permeability [145]. It was reported that the permeation of vitamin B6 through a chicken pouch mucosa from its control solution was 1.5 times higher than that from the control film. The permeation was 1.2 times higher for standalone PEG-liposomes as a dispersion in comparison to the PEG-liposomes in the film counterpart. This indicates that the system provides a slower and prolonged release. It was also noticed that both films showed quicker initial permeation than their liquid counterparts, which the authors attributed to better contact with the mucosa. Nevertheless, PEG-liposomes in films showed better permeability than the control film, and similarly, standalone PEG-liposomes performed better than the control solution. Similarly, Maura et al. incorporated PEG-liposomes into a mucoadhesive gel that aimed to deliver a peptide (opiorphin) through the nasal mucosa [148]. An *ex vivo* permeation study of the PEG-liposomes-in-gel formulation showed great permeation of around 60% at the 5 h time point, whereas the standalone gel showed a 2 h delay, and only around 10% permeated within the same timeframe, most likely due to poor diffusion of opiorphin through the hydrogel.

An interesting approach was proposed by Li Wei-Ze et al., who designed mucoadhesive hydrogel foam aerosols loaded with PEG-liposomes that, upon application in the vaginal cavity, expand, providing great coverage and close contact [141]. The presence of PEG-liposomes did not have any noticeable effect on the mucoadhesive properties of the hydrogel foams. However, a surprising finding was reported in this study. Drug permeation *in vitro* through porcine vaginal mucosa was over 3-fold higher for the expansile hydrogel foam aerosol in comparison to the standalone hydrogel. Moreover, PEG-liposomes loaded into the mucoadhesive hydrogel foam aerosol showed the highest permeation, followed by PEG-liposomes in the foam aerosol without mucoadhesive properties. The post-expansile hydrogel foam aerosol caused stretching of the mucosa, thus increasing its pore sizes, which, in turn, allowed for easier penetration of PEG-liposomes, and this effect was strengthened by the mucoadhesive properties of the hydrogel, further increasing permeation.

The study conducted by Freag et al. should be emphasized as it provides a better understanding of the efficacy that this delivery strategy offers [144]. They prepared phytosome nanocarriers (PHY) with tripterine drug, coated their surface with positively charged protamine, and incorporated them into mucoadhesive sponges. Through control of the protamine amount, three different phytosomes with cationic, near-neutral, and negative surface charges were obtained. Cationic PHY increased the mucoadhesive strength of sponges in comparison to negatively charged PHY and blank sponges. Ethyl cellulose was used as a backing layer of the sponges (lamination) to provide unidirectional release, and its presence was also evaluated. *In vitro* release studies showed that lamination decreased the amount of drug released from 20% to 8.6% for negatively charged PHY-in-sponges after 6 h. Overall release from sponges coated with PHY was slower in comparison to uncoated sponges due to possible interactions between protamine and chitosan. Although the authors did not highlight it, PHY with near-neutral charge in unlaminated sponges showed greater release *in vitro* after 6 h, whilst laminated sponges with positively charged phytosomes released more drugs in comparison to near-neutral ones. The permeation of standalone PHY through chicken pouch mucosa was the lowest for negatively charged PHY, followed by near-neutral PHY, and the highest was obtained for cationic PHY. Interestingly, near-neutral and cationic PHY showed similar permeation initially, but the authors attributed the final decrease in the former to the detachment of protamine due to its low concentration. For PHY-in-sponges, it was shown that lamination increased permeation *in vitro* due to its unidirectional release. *In vivo* buccal release in rabbits showed that cationic PHY-in-sponge had 240% higher bioavailability in comparison to uncoated PHY-in-sponge; moreover, the drug was still detectable in serum after 3 h for coated phytosomes, but not for uncoated ones.

A second study that is worth exploring in more detail was conducted recently by Rosso et al., who combined mucodiffusive nanoemulsions into chitosan sponges [147]. A very in-depth analysis of nanoemulsion mucodiffusive properties was conducted using different methods, and no significant interaction with mucin was observed. Although mucoadhesive studies were not included, the water uptake of sponges decreased after the addition of nanoemulsions, and an increase in stiffness was also observed, thus indicating interaction between nanoemulsions and sponges. Most importantly, the biodistribution and transit of chitosan–nanoemulsion sponges in the gastrointestinal tract of mice were studied and compared with standalone nanoemulsions and chitosan–nanoemulsion mixtures at different time points. After 6 h, nanoemulsions were detected mostly in the rectum, meaning they traversed without obstruction. A mixture of nanoemulsions and chitosan slowed down the transit of the system and thus prolonged the residence time. At 6 h, a fluorescent signal was detected mostly in the colon and cecum, but not in the rectum. Nanoemulsions in chitosan sponges significantly improved intestinal residence time, and the highest prevalence was detected in the cecum at the 6 h time point. However, due to the quenching effect, fluorescent intensity was underestimated in the case of nanoemulsions in chitosan sponges, as noted by the authors.

In contrast to other macrostructures, electrospun fibers possess a high surface-to-volume ratio, great flexibility, and are highly porous. All these properties promote the formation of intimate contact with the mucosa. Krogstad et al. proposed a system composed of water-soluble, mucoadhesive PVA or PVP electrospun fibers and incorporated into them mucodiffusive PEG-PLGA NPs at the stage of electrospinning solution preparation so that the NPs were enclosed within individual fibers (PVA-NPs and PVP-NPs) [149]. They showed that both PVA and PVP fibers dissolved within 30 min *in vitro* under sink conditions. However, the situation changed *in vivo*, and even after 24 h, undissolved fibers were still present on the mucosa. Although PVA-NP fibers could be easily washed away with a buffer, PVP-NP fibers remained attached to vaginal tissue due to their stronger mucoadhesion. Both types of fibers prevented leakage of encapsulated NPs from the vaginal cavity in comparison to aqueous suspensions of NPs. After 24 h, it was shown that PVA-NPs fibers provided over 30 times higher retention of NPs on the mucosa and increased delivery by 22-fold to vaginal tissue; however, for the PVP-NPs, this could not be precisely evaluated due to the residual presence of fibers on the mucosa that could not be washed away, and only PVA fibers were included in further studies. After 48 h and 72 h, PVA-NPs fibers retained around 47% and 38% of the total dosage in the vaginal cavity, respectively, whereas after 120 h, the dosage was barely detectable. In comparison, aqueous suspensions retained only 1.5% of the dosage after 24 h. Despite the great results obtained with rhodamine-loaded NPs, a crucial comparison between doses in cervicovaginal lavage and vaginal tissue was included for NPs with a lipophilic drug (etravirine) classified as BCS IV. Release in vaginal lavage showed burst release from PVA-NP fibers, reaching maximal concentration at the 1 h time point, similar to NP suspensions, but the maximal drug concentration was significantly higher for PVA-NPs (84% of total dosage) in comparison to NP suspensions (17% of total dosage). On the other hand, such burst release was not observed during the evaluation of the drug concentration in the vaginal tissue. A two-fold increase in drug concentration in the vaginal tissue was observed for PVA-NP fibers between 24 and 72 h, whereas bioavailability was around 23% higher than for suspensions. Analysis of secondary organs to drug exposure showed very low overall drug concentrations, but it was significantly higher for PVA-NP fibers than suspensions, thus indicating improved permeability. Although the dose in the vaginal tissue was only slightly higher for PVA-NP fibers in comparison to NP suspensions, this system provided much better prolonged release. We speculate that if the issue of NP burst release in the lavage could be resolved, for example, by including a backing layer, a significantly higher bioavailability in the vaginal tissue could be achieved in similar systems.

A more general approach to studying HMDSs based on electrospun fibers was reported by Mašek et al., who designed a three-layer composite of a nanofibrous scaffold containing freely dispersed nanocarriers, a mucoadhesive film, and a backing layer [150]. In their work, they evaluated three different materials for nanofibrous scaffolds, i.e., silk fibroin (SF), chitosan–polyethylene oxide (CS-PEO), and polycaprolactone (PCL). They also studied hybrid systems with PEG-PLGA and PEG-liposomes separately. The use of the Cryo-SEM method confirmed that the nanofibrous depot formed very intimate contact with the porcine sublingual mucosa, which protects it from salivary flow. All hydrophilic fibers (SF, CS-PEO) released both types of nanocarriers within 30 min, except for hydrophobic PCL, which released 50% within the same timeframe. However, pretreatment to improve the hydrophilic properties of PCL resulted in complete release, similar to other fibers, indicating the crucial effect of fiber hydrophilicity on nanocarrier release. Moreover, *ex vivo* studies on porcine oral and sublingual mucosa showed that standalone nanocarriers were quickly washed out and showed no penetration at all, whereas nanofibrous composites allowed for their diffusion into the tissues. The authors provided much more valuable information showing the distribution of nanocarriers in mucosae, coupled with insightful comments on parameters affecting the performance of such systems, but it will not be discussed here in more detail. Nevertheless, this article is a “must-read” for anyone planning to design an HMDS of nanocarriers-in-fibers in the future.

### 5.3. Mucolytic Nanocarriers

Mucolytic nanocarriers are generally less popular in the literature than the other two mucointeractive strategies. It is not surprising, then, that the combination of these nanocarriers with mucoadhesive macrostructures is less researched. Nevertheless, a few reports can be found. One group provided three consecutive articles about such systems that aimed to deliver insulin through the buccal mucosa. In the first study, thiolated dimethyl ethyl chitosan (DMEC) nanoparticles were prepared and incorporated into chitosan films [152]. A comparison of *in vitro* release between chitosan NPs, DMEC NPs, and thiolated DMEC NPs showed that 72, 82, and 98% of insulin was released after 8 h. The relationship was in accordance with an *ex vivo* permeation study through rabbit buccal mucosa that confirmed the highest permeation of thiolated DMEC. The other two articles focused mainly on the use of response surface methodologies to optimize buccal films with mucolytic NPs [153,154]. On the other hand, a different study incorporated native papain and nanopapain into mucoadhesive hydrogels for the delivery of gemcitabine for bladder cancer treatment [81]. Both the native enzyme and its nanoparticulate form improved permeation through bladder tissue, but the use of the native papain resulted in quicker permeation. It must be noted that in this study, the drug was dispersed within the hydrogel and not encapsulated into a separate carrier, like in previous examples.

Although results are promising, based solely on this data, it is difficult to evaluate the efficacy of mucolytic nanocarriers in the mucoadhesive macrostructures approach, and more studies are required.

## 6. Conclusions and Future Outlook

In summary, basic mucointeractive strategies (i.e., mucoadhesion, mucodiffusion, mucolysis) improve mucosal drug delivery, but each of them has its own disadvantages. The barrier-like properties of mucus are only partially addressed by these three strategies, and for this reason, mucosal drug delivery cannot be utilized to its full potential. Development of HMDSs seems like the next logical step forward to overcome the mucosal barrier. As of now, two main types of HMDSs can be distinguished: particulate two-in-one HMDSs and particles-in-macrostructure HMDSs.

Particulate two-in-one HMDSs combine two basic mucointeractive strategies in a single system in the form of nano- or microparticulate carriers. These HMDSs address the limitations of basic mucointeractive strategies. Zeta potential-changing systems are initially capable of great coverage due to their mucodiffusive properties and switch their surface charge to positive in a predictable manner due to specific stimuli. Such systems could be used for the delivery of drugs that show poor permeability in mucus and poor cellular uptake. Mucoadhesion-to-mucodiffusion HMDSs, due to their particulate nature, provide great coverage upon application, whilst mucoadhesion limits their further spread and provides localized release. For example, the use of such a system in a mouthwash could allow particles to adhere in the oral cavity and potentially limit the amount of drug that could reach the gastrointestinal tract. Mucoadhesion–mucolysis HMDSs act similarly, but in these systems, mucolysis increases mucus permeability, while mucoadhesion prevents their further spread and prolongs residence time. Based on the available studies, we conclude that particulate two-in-one HMDSs might be better at delivering therapeutics for topical treatments of mucosal-related diseases.

On the other hand, particles-in-macrostructure HMDSs presented sustained delivery and great depth of penetration into mucosa and submucosa, but such systems might be too localized for the treatment of mucosal diseases. For this reason, these systems might be more suitable as a non-invasive alternative to subcutaneous systemic delivery or delivery to underlying tissues. High localization does not necessarily disqualify them as a possible treatment of local afflictions if designed correctly, as discussed previously in the cases of expansile foams and in situ hydrogels that provided great coverage [141]. It must be noted, however, that their use in topical delivery to the mucosa might cause an unnecessary exposure of therapeutics to secondary organs and systemic circulation. At this point in time, it cannot be clearly concluded whether mucoadhesive, mucodiffusive, or mucolytic nanocarriers provide the best permeation through the mucosa once released from a mucoadhesive macrostructure. What can be stated is that both mucoadhesive and mucodiffusive nanocarriers showed better mucosal penetration once incorporated into a macrostructure in comparison to standalone nanocarriers. Moreover, PEGylated nanocarriers are also referred to as stealth nanocarriers and particles-in-macrostructure HMDSs, and such nanocarriers could potentially perform much better in systemic delivery of drugs. On the other hand, mucoadhesive nanocarriers may interact with physiological components once in circulation; thus, their systemic exposure could be lower, and they might be removed more quickly from the organism. This phenomenon could be used in applications where conventional drug delivery systems fail to achieve sufficient depth of penetration, such as delivery to the submucosa or even deeper layers, while simultaneously minimizing dosage in systemic circulation. However, these considerations require further studies (Table 3).

The articles discussed in this review that presented HMDSs had sometimes different goals; however, it must be highlighted that several of the studies, especially those discussed in Section 5, did not use sufficiently expanded methodologies to properly evaluate the efficacy of the tested HMDSs. Here, we propose a similar approach to that undertaken, for example, by Shawky et al. [138]. Both nanocarriers and macrostructures should be evaluated separately and compared with a system that combines them in all tests, if possible. We recommend that future studies include mucointeractive studies of each component, as well as mucus permeation and/or mucosal penetration depth of nanocarriers and nanocarriers released from macrostructures. Such a methodology will allow for the proper evaluation of such particles-in-macrostructure HMDSs, at least to a minimal extent.

Furthermore, it must be noted that the delivery systems discussed in this work were evaluated in preclinical conditions. The performance of drug delivery systems may differ significantly in clinical trials. A recent review article provided an insight into the clinical translation of nanoparticle-loaded hydrogels [155]. The authors concluded that 20 clinical studies are in progress. However, due to inconsistent terminologies in some of these studies, they could not clearly define whether an actual hydrogel/gel was used or just a viscous solution. It is worth noting that these authors focused on systems for precision medicine, which is a significantly wider scope than the systems discussed in this work. This especially shows the novelty of HMDSs and how little is known about their clinical performance. On the other hand, such systems may face complex obstacles before being available on the market. One such limitation might be their stability and shelf-life due to their biodegradable or bioabsorbable nature. Moreover, issues with scalability for mass production must be considered, such as homogenous distribution of nanoparticles within macrostructures and batch-to-batch differences in their properties.

Additionally, nanoparticulate systems in general are well researched, and several drug delivery systems are already approved by the FDA. However, it must be noted that nanoparticulate two-in-one HMDSs differ in their complexity, and obtaining proper approval might prove challenging. The situation might be even more difficult for particles-in-macrostructure HMDSs, in which two components must be first evaluated separately and then together. This poses a serious challenge for commercialization of HMDSs that will require extensive studies. Recent advances in the use of AI in the field of drug delivery might prove helpful in overcoming some of these hurdles [156].

Nonetheless, available studies on HMDSs show their great potential in mucosal delivery, and in many cases, these systems significantly outperform standalone mucointeractive delivery systems. However, their great performance comes from their complexity. These HMDSs require more modifications, more components, and/or more steps in their preparation. Considering large-scale production, this limitation might prove costly. Whether HMDSs will find their way into the pharmaceutical market remains to be seen. For the time being, future studies will most likely try to further improve their mucosal delivery efficacy, but more focus might also be directed towards a better understanding of synergies between multiple mucointeractive strategies within one system.

## Figures and Tables

**Table 1:** Mucoadhesive nanocarriers in mucoadhesive macrostructures.

Mucoadhesive Nanocarrier	Mucoadhesive Macrostructure	Target Mucosa	Testing Method	Test Duration	Ref.
Chitosan NPs	HPMC-based film	Buccal	*In vivo* (rats)	5 h	[128]
Chitosan NPs	HPMC/poly(acrylic acid) film	Buccal	*In vitro*	7 h	[129]
Chitosan NPs	HPMC film	Ocular	*In vivo* (rabbits)	12 h	[130]
Chitosan-coated PCL NPs	Chitosan film	Buccal	*In vitro*	24 h	[131]
Chitosan/sodium alginate NPs	Sodium alginate film	Buccal	*In vitro*	24 h	[132]
Carboxylation—chitosan NPs	Chitosan–ethylenediaminetetraacetic acid film	Gastrointestinal	*In vivo* (rats)	3 h	[133]
Zein NPs	Sodium alginate film	Buccal	-	-	[134]
Chitosan-coated lipid-core nanocapsules (LNCs)	HPMC/PEG-PPG copolymer gel	Buccal	*In vitro*	8 h	[135]
Folate-conjugated chitosan–lipidic nanoparticles	Poly(acrylic acid) gel	Buccal	*Ex vivo*	8 h	[136]
Chitosan-coated liposomes	Poly(acrylic acid) gel	Buccal	*In vivo* (rats)	30 days	[137]
Chitosan-coated solid lipid nanoparticles	PEG-PPG copolymer-based gels	Bladder	*Ex vivo*	4 h	[138]

**Table 2:** Mucodiffusive nanocarriers in mucoadhesive macrostructures.

Mucodiffusive Nanocarrier	Mucoadhesive Macrostructure	Target Mucosa	Testing Method	Test Duration	Ref.
PEG-lipoplexes ^a^	Sodium alginate sponge	Vaginal	*In vivo* (mice)	24 h	[139]
Deformable PEG-liposomes	Poly(acrylic acid) hydrogel	Vaginal	*In vitro*	24 h	[140]
PEG-liposomes	Hydroxyethyl cellulose hydrogel foam	Vaginal	*In vitro*	12 h	[141]
PEG-lipoplexes	Hydroxyethyl cellulose sponge	Vaginal	*In vitro*	6 h	[142]
Deformable PEG-liposomes	Chitosan hydrogel	Vaginal	*In vitro*	28 days	[143]
Protamine-coated phytosomes	Chitosan–hydroxypropyl methylcellulose sponges	Buccal	*Ex vivo*	6 h	[144]
PEG-liposomes	Hydroxypropyl methylcellulose–carboxymethylcellulose sodium film	Buccal	*Ex vivo*	6 h	[145]
PEG-*b*-PLA nanoparticles	Chitosan film	Buccal	*In vitro* (only NPs)	45 days	[146]
Nanoemulsions	Chitosan sponge	Oral	*In vivo* (mice)	6 h	[147]
PEG-liposomes	PEG-PPG copolymer/poly(acrylic acid) gel	Nasal	*Ex vivo*	5 h	[148]
PEG-PLGA	PVA and PVP electrospun fibers	Vaginal	*In vivo* (mice)	7 days	[149]
PEG-PLGA and PEG-liposomes	Multi-layered electrospun fibers composite	Oral	*In vivo* (piglets)	2 h	[150]

^a^ Nonviral lipid carrier of DNA, composed of cationic liposomes that form complexes with DNA.

**Table 3:** Comparison of HMDSs from the standpoint of their respective opportunities and challenges.

HMDS	Opportunities	Challenges
Zeta potential-changing systems	- Great coverage due to small size. - Specific stimuli induce charge change (targeted delivery). - Promotes cellular uptake (good depth of penetration). - Dozens of research articles studying such systems. - Potential applications: systemic and topical delivery.	- Back diffusion. - Difficulties with obtaining satisfactory differences in positive/negative charges.
Mucoadhesion-to-mucodiffusion systems	- Quick initial immobilization of nanocarriers. - Localized release with limited spread of therapeutics. - Potential application: topical delivery.	- Few research articles.
Mucolytic mucoadhesive systems	- Great initial immobilization of nanocarriers. - Depth of penetration improved through mucolysis. - Potentially high concentration of therapeutics attainable in localized spot. - Potential application: topical delivery.	- Mucolysis-related side effects. - Few research articles.
Mucoadhesive nanocarriers in mucoadhesive macrostructures	- Highly localized release of nanocarriers. - Mucoadhesive nanocarriers released close to epithelium. - Prolonged residence times. - Greater control over release kinetics than standalone nanocarriers by shaping macrostructure properties. - Potential applications: systemic and topical delivery that require good penetration depth.	- Such complex systems may face difficulties with FDA approval and commercialization. - Stability of nanocarriers in the macrostructure. - Few research articles.
Mucodiffusive nanocarriers in mucoadhesive macrostructures	- Highly localized release of nanocarriers. - May prevent back diffusion of mucodiffusive nanocarriers. - Greater control over release kinetics than standalone nanocarriers by shaping macrostructure properties. - Potential application: mainly systemic delivery.	- Such complex systems may face difficulties with FDA approval and commercialization. - Stability of nanocarriers in the macrostructure. - Few research articles.
Mucolytic nanocarriers in mucoadhesive macrostructures	- Highly localized release of nanocarriers. - Mucolysis may allow for a quick diffusion of nanocarriers towards epithelium. - Greater control over release kinetics than standalone nanocarriers by shaping macrostructure properties. - Potential applications: quick-acting systemic and topical delivery.	- Mucolysis-related side effects. - Such complex systems may face difficulties with FDA approval and commercialization. - Stability of nanocarriers in the macrostructure. - Few research articles.

## Data Availability

No new data were created or analyzed in this study. Data sharing is not applicable.
